# Validation of the "World Health Organization Disability Assessment Schedule, WHODAS-2" in patients with chronic diseases

**DOI:** 10.1186/1477-7525-8-51

**Published:** 2010-05-19

**Authors:** Olatz Garin, Jose Luis Ayuso-Mateos, Josué Almansa, Marta Nieto, Somnath Chatterji, Gemma Vilagut, Jordi Alonso, Alarcos Cieza, Olga Svetskova, Helena Burger, Vittorio Racca, Carlo Francescutti, Eduard Vieta, Nenad Kostanjsek, Alberto Raggi, Matilde Leonardi, Montse Ferrer

**Affiliations:** 1 Health Services Research Unit, IMIM-Hospital del Mar, Barcelona, Spain; 2 CIBER en Epidemiología y Salud Pública (CIBERESP), Spain; 3 Department of Psychiatry, Hospital Universitario de la Princesa. Universidad Autonoma de Madrid; CIBERSAM, Madrid, Spain; 4 Health Statistics and Informatics, WHO, Geneva, Switzerland; 5 Ludwig-Maximilians-University, Munich, Germany; 6 Charles University, Prague, Czech Republic; 7 Institute for Rehabilitation, Ljubljana, Slovenia; 8 Fondazione Don Carlo Gnocchi- Onlus, Milano, Italy; 9 Agenzia Regionale Sanità, Pordenone, Italy; 10 Hospital Clinic, University of Barcelona, IDIBAPS, CIBERSAM, Barcelona, Spain; 11 Fondazione IRCCS, Instituto Neurologico Carlo Besta, Milano, Italy; 12 Universitat Autònoma de Barcelona, Spain

## Abstract

**Background:**

The WHODAS-2 is a disability assessment instrument based on the conceptual framework of the International Classification of Functioning, Disability, and Health (ICF). It provides a global measure of disability and 7 domain-specific scores. The aim of this study was to assess WHODAS-2 conceptual model and metric properties in a set of chronic and prevalent clinical conditions accounting for a wide scope of disability in Europe.

**Methods:**

1,119 patients with one of 13 chronic conditions were recruited in 7 European centres. Participants were clinically evaluated and administered the WHODAS-2 and the SF-36 at baseline, 6 weeks and 3 months of follow-up. The latent structure was explored and confirmed by factor analysis (FA). Reliability was assessed in terms of internal consistency (Cronbach's alpha) and reproducibility (intra-class correlation coefficients, ICC). Construct validity was evaluated by correlating the WHODAS-2 and SF-36 domains, and comparing known groups based on the clinical-severity and work status. Effect size (ES) coefficient was used to assess responsiveness. To assess reproducibility and responsiveness, subsamples of stable (at 6 weeks) and improved (after 3 moths) patients were defined, respectively, according to changes in their clinical-severity.

**Results:**

The satisfactory FA goodness of fit indexes confirmed a second order factor structure with 7 dimensions, and a global score for the WHODAS-2. Cronbach's alpha ranged from 0.77 (self care) to 0.98 (life activities: work or school), and the ICC was lower, but achieved the recommended standard of 0.7 for four domains. Correlations between global WHODAS-2 score and the different domains of the SF-36 ranged from -0.29 to -0.65. Most of the WHODAS-2 scores showed statistically significant differences among clinical-severity groups for all pathologies, and between working patients and those not working due to ill health (p < 0.001). Among the subsample of patients who had improved, responsiveness coefficients were small to moderate (ES = 0.3-0.7), but higher than those of the SF-36.

**Conclusions:**

The latent structure originally designed by WHODAS-2 developers has been confirmed for the first time, and it has shown good metric properties in clinic and rehabilitation samples. Therefore, considerable support is provided to the WHODAS-2 utilization as an international instrument to measure disability based on the ICF model.

## Background

A common, international, and interdisciplinary framework of disability measurement is important to develop effective and comparable policy and practice options[1,2]. During the last decades, the definition of disability has moved from the biomedical and social models to the biopsychosocial model, emphasizing the dynamic and bidirectional relations between a health condition and contextual factors (personal and environmental). In order to reach a universally accepted conceptual framework to define and classify disability[3,4], the World Health Organization (WHO) developed the International Classification of Functioning, Disability, and Health (ICF)[5,6]. In the ICF, disability is described as *"a difficulty in functioning at the body, person, or societal levels, in one or more life domains, as experienced by an individual with a health condition in interaction with contextual factors"*[7].

As part of the ongoing development of the ICF conceptual model, the World Health Organization Disability Assessment Schedule 2.0 (WHODAS-2) was created in 1998 (as a substantially reviewed version of the WHO-DAS[8]) to assess disability based on the ICF model[9]. There exist other tools that have traditionally been used to measure disability, such as the Indexes of activities of daily living (ADLs)[10], the Functional Limitations Profile[11], or the Functional Status Questionnaire[12]; and also a battery of instruments developed focusing on specific populations (i.e., the Late Life Function and Disability Instrument for elders[13], and the Functional Disability Inventory for children[14]). Nevertheless, none of them has been developed with the clear ICF biopsychosocial conceptual model.

Previous studies have evaluated the metric properties of the WHODAS-2 in specific samples, such as arthritis[15], systemic sclerosis[16], psychotic disorders[17], hearing loss[18], stroke[19], ankylosing spondylitis;[20], depression and low back pain[21], schizophrenia[22], and patients in rehabilitation[23], among others[24]. However, data regarding the validity of the WHODAS-2 across a range of diagnoses, settings, and countries is missing. On the other hand, these studies were generally focused on reliability, validity or responsiveness, but the underlying factor structure has almost never been assessed. Available evidence confirming the original structure is only provided for a modified version (i.e. the WHODAS used in the WMH surveys initiative[25,26]), while findings from WHODAS-2 exploratory factor analysis were not consistent with the proposed measurement model [23,24]. Thus, a comprehensive evaluation of the conceptual model and metric properties of the WHODAS-2 is needed.

The 'Measuring Health and Disability in Europe: Supporting policy development-MHADIE'[8,27] is a European multidisciplinary project which has as one of its main objectives the evaluation of the ICF model and related instruments in clinical and rehabilitative settings. As part of this international project, the aim of the present study was to assess the WHODAS-2 conceptual model and metric properties in a set of chronic and prevalent clinical conditions, both physical and mental disorders, accounting for a wide scope of disability in Europe.

## Methods

### Design

The MHADIE is an observational, longitudinal, multicentric study of consecutive patients with different chronic conditions in 7 European centres from Czech Republic, Germany, Italy, Slovenia, and Spain. Evaluations were made at baseline and at 6 weeks and 3 months of follow-up. Background characteristics such as age, sex, education or occupational status were collected from all subjects. In addition, patients were clinically evaluated with disease-specific severity scales, and with standardised instruments measuring disability and quality of life.

### Sample

Patients had to be over 18 years old and meet the diagnosis criteria of one of the following conditions: bipolar disorder, depression, osteoarthritis, osteoporosis, rheumatoid arthritis, chronic widespread pain (CWP), low back pain (LBP), ischemic heart disease (IHD), migraine, Parkinson disease, multiple sclerosis, traumatic brain injury (TBI), or stroke. Sample size was based on recommendations for exploratory and confirmatory factor analyses (at least 20 participants per variable), and balanced by disorder. Ethical approvals from each institutional ethics committee and informed consent from each participant were obtained.

### Measurement instruments

#### The World Health Organization Disability Assessment Schedule-2

The WHODAS-2 contains 36 items on functioning and disability with a recall period of 30 days[8] covering 7 domains: *Understanding and Communicating (6 items), Getting around (5 items)*, *Self-care (4 items)*, *Getting along with others (5 items)*, *Life activities: household (4 items), Life activities: work/school (4 items)*, and *Participation in society (8 items)*. Response options go from 1 (no difficulty) to 5 (extreme difficulty or can not do).

WHODAS-2 scores are computed for each domain by adding the item responses (the score computation allows for up to 30% of missing items per domain) and transforming them into a range from 0 to 100, with higher scores indicating higher levels of disability. A global score is also calculated from all the items (36) or from all except the Life activities ones -work/school- when people does not apply for this domain (32 items). When less than 50% of items were missing, mean substitution (by domain) was used for imputation.

#### The Short Form-36 Health Survey (SF-36)

The SF-36 is a generic Health Related Quality of Life (HRQL) instrument measuring 8 domains: *Physical Functioning, Role Physical, Bodily Pain, General Health, Vitality, Social Functioning, Role Emotional*, and *Mental Health*[28]. Items are transformed into scores from 0 (worst possible health state) to 100 (best). A weighted addition of these domains allows the computation of two summary scores: Physical and Mental Components Summaries (PCS & MCS)[29,30]. Scores were not computed for those individuals with more than 50% of missing items per domain. All patients were administered the SF-36 version 1, except those with bipolar disorder or depression, that completed version 2. Main differences between the two versions concern the number of response options of the Role domains, which were incremented from 2 to 5; and minor changes in the mental health and vitality dimensions (from 6 to 5 response options)[31].

#### Disease-specific severity scales

As shown in Table 1, several different scales were used to evaluate the severity of the health conditions [32-40]. A consensus on the best way of classifying patients into different severity groups in order to evaluate differences on WHODAS-2 scores was reached between researchers and the clinical specialist responsible of the patients' management. Criteria used for classifying patients as being mild, moderate or severe are defined in Table 1. The sample sizes of the final groups are also shown.

**Table 1:** Health condition, severity scales and criteria to make groups.

Health Condition	Severity Scales	Theoretical Range	Groups' criteria	Severity Groups, n
Bipolar Disorder	*Young Rating Scale of Mania *(YRSM) &*Hamilton Depression Rating Scale *(HDRS)	YRSM_ 0-60 HDRS_ 0 - 52	Eutimic if YRSM < 7 and HDRS < 9	Eutimic, 78 No eutimic, 36
Depression	*International Classification * *of Disease (ICD-10)*	__	Clinicians criteria	Mild, 36 Moderate, 30 Severe, 19
Osteoarthritis	---	---	---	---
Osteoporosis	*Magnitude of the * *problems in functioning*	0-10	Terciles (0-1) (2-3) (4-10)	Mild, 41 Moderate, 27 Severe, 17
Rheumatoid Arthritis	*Criteria for the * *Classification of Global * *Functional Status *(ACR)	Class I-IV	I, II-III, and IV	Mild, 5 Moderate, 16 Severe, 2
Chronic Widespread Pain (CWP)	*Pain intensity rate*	0 - 10	Terciles (0-4) (5-6) (7-10)	Mild, 15 Moderate, 14 Severe, 13
Low Back Pain (LBP)	*Pain intensity rate*	0 - 10	Terciles (0-3) (4-6) (7-10)	Mild, 42 Moderate, 44 Severe, 24
Ischemic Heart Disease (IHD)	*New York Heart * *Association Criteria * (NYHA)_ IV classes	Class: I-IV	I II III	Mild, 12 Moderate, 71 Severe, 17
Migraine	*Migraine Disability * *Assessment * *Questionnaire *(MIDAS)	4 groups	Minimal-mild, moderate, and severe	Mild, 27 Moderate, 29 Severe, 46
Parkinson Disease	*Hoehn and Yahr scale * (H&Y)_5 groups	5 stages	1, 2, and ≥ 3	Mild, 13 Moderate, 54 Severe, 26
Multiple Sclerosis	*Expanded Disability Status* * Scale *(EDSS)	0-10	(0-2.5) (3-5) (5.5-10)	Mild, 43 Moderate, 36 Severe, 21
Traumatic Brain Injury (TBI)	*Functional Independence * *Measure *(FIM)	18 - 126	< 116; 116-126; and ≥ 126	Mild, 36 Moderate, 33 Severe, 31
Stroke			< 47; 47-63; and ≥ 63	Mild, 78 Moderate, 24 Severe, 2^†^

^†^Excluded from analyses as for most WHODAS-2's scores information for just one of the two individuals was available.

Questionnaires were either self-administered or interviewer-administered. Proxy versions were occasionally used with those patients unable to respond due to the severity of the health condition leading to cognition or communication difficulties, such as aphasia.

### Analytical strategy

Exploratory and Confirmatory factor analyses (EFA & CFA) were performed to assess WHODAS-2 structure and dimensionality. The global sample at baseline was divided into two random sub-samples, stratifying by pathology and severity group (n_1 _= 533 and n_2 _= 547). As WHODAS-2 responses are categorical variables, the factorial analyses were based on polychoric correlations, and robust-weighted least squares estimators were used[41,42]. The first subsample (n_1_) was used to perform an EFA with oblique (quartimin) rotation[43]. The factor structure obtained by the EFA was assessed on the CFA using the second subsample (n_2_). The model to be confirmed was also imposed to have a general (global) second order factor; related with the specific factors. On this type of models, the general factor (2^nd ^level) explains the correlation among specific factors (first level)[44]. Goodness-of-fit was measured by the Root Mean Square Error of Approximation (RMSEA, adequate if below 0.08), and the Comparative Fit Index (CFI) and Tucker-Lewis Index (TLI), which are recommended to be over 0.95[45]. These analyses were conducted with MPlus 4.2 and missing values were considered missing at random[45].

Distribution of WHODAS-2 and SF-36 scores was evaluated for the whole sample: means (SD), observed range, percentage of patients with missing domain scores, and floor and ceiling effects (proportion of patients with the worst and best possible score, respectively). Reliability was assessed in terms of internal consistency and reproducibility. The former was evaluated with the Cronbach's alpha coefficients computed with the whole sample at baseline[46]. To assess reproducibility, a sub-sample of stable patients (their clinical-severity not having changed at the six weeks evaluation) was identified. Concordance in the scores of stable patients was estimated with the Intra-class Correlation Coefficient (ICC)[47].

Construct validity was assessed by 2 different approaches: the Multitrait Multimethod (MTMM) Matrix[48] and known groups. Taking into account similarity on content, Pearson correlations (MTMM) were previously hypothesized to be moderate (0.4-0.6) between some of the WHODAS-2 domains and the SF-36 scores. Known groups were defined in two ways: first, based on the severity of the health condition (mild, moderate, and severe) and second, based on whether the patients were working or not due to their health condition (i.e. those who were on sick leave or who reported "ill health" as the main reason for not working for pay). Means scores were compared with ANOVA and the magnitude of the difference between extreme groups was measured by an Effect Size coefficient (difference in mean scores between groups/pooled SD)[49].

To assess sensitivity to change, the only conditions included were those where an improvement was expected over the study period (all except bipolar disorder, osteoarthritis, Parkinson disease, and multiple-sclerosis). Patients suffering from any of these pathologies with a positive change in the severity measure after 3 months were considered "clinically improved". Paired mean comparisons (t-test) between baseline and the third evaluation of these patients were conducted. In this case, the magnitude of the difference was also assessed with ES coefficients, but computed dividing the difference in the scores between the two evaluations by the SD at baseline. An ES > 0.8 is considered high, one of 0.5 moderate, and one close to 0.2 is considered low[50].

## Results

Sample characteristics are shown in Table 2. More than half of the subjects were not working for pay (57.8%), and 49% of them (n = 327) reported a main reason: 184 retired and 75 with 'ill health'. The EFA showed the 7-factor model to be the most appropriate structure (Table 3). Most of the WHODAS-2 items (86%) presented the highest loading with their corresponding factor. Moreover, the highest factor loadings of each item was above 0.5 in 75% of the cases. Results of CFA presented acceptable goodness of fit indexes: CFI and TLI above the standard 0.95 (0.975 and 0.973), and RMSEA (0.127); and supported the 7 domains proposed, as well as the global score.

**Table 2:** Socio-demographic characteristics of global sample, and the reproducibility and improvement sub-samples.

	All patients baseline, n = 1190	Stable at 6 weeks, n = 404	Improved at 3 months, n = 131
**Sex, n (%)**		*	*
Male	520 (43.8%)	205 (50.7%)	45 (34.4%)
Female	666 (56.2%)	199 (49.3%)	86 (65.6%)
**Age, mean (SD)**			
	52.7 (15.6)	53.4 (16.0)	54.5 (14.5)
**Marital status, n (%)**			
Never married	233 (20.0%)	71 (17.9%)	20 (15.6%)
Currently married	659 (56.6%)	247 (62.4%)	68 (53.1%)
Separated	40 (3.4%)	7 (1.8%)	4 (3.1%)
Divorced	73 (6.3%)	22 (5.6%)	14 (10.9%)
Widowed	92 (7.9%)	30 (7.6%)	14 (10.9%)
Cohabiting	68 (5.8%)	19 (4.8%)	8 (6.3%)
**Highest level of education, n (%)**		*	
No formal schooling	4 (0.4%)	1 (0.3%)	1 (0.8%)
Less than primary school	28 (2.5%)	5 (1.3%)	4 (3.2%)
Primary school completed	241 (21.9%)	90 (22.8%)	39 (31.5%)
Secondary school completed	256 (23.2%)	106 (26.9%)	39 (31.5%)
High school (or equivalent) completed	281 (25.5%)	108 (27.4%)	16 (12.9%)
College/University completed	266 (24.1%)	79 (20.1%)	23 (18.5%)
Postgraduate degree completed	26 (2.4%)	5 (1.3%)	2 (1.6%)
**Current job, n (%)**		*	
Government employee	132 (11.5%)	43 (11.1%)	20 (15.4%)
Non-government employee	221 (19.3%)	69 (17.8%)	26 (20.0%)
Self-employed	99 (8.6%)	37 (9.6%)	13 (10.0%)
Employer	32 (2.8%)	11 (2.8%)	2 (1.5%)
Not working for pay	663 (57.8%)	227 (58.7%)	69 (53.1%)
**Health conditions, n (%)**		*	
Bipolar	114 (9.6%)	4 (1.0%)	---
Depression	83 (7.0%)	15 (3.7%)	19 (14.5%)
Musculo-skeletal conditions	297 (25.0%)	57 (14.1%)	27 (20.6%)
Osteoarthritis	19 (1.6%)	---	---
Osteoporosis	87 (7.3%)	17 (29.8%)	4 (14.8%)
Rheumatoid Arthritis	24 (2.0%)	11 (19.3%)	3 (11.1%)
Chronic Widespread Pain (CWP)	49 (4.1%)	12 (21.1%)	9 (33.3%)
Low Back Pain (LBP)	118 (9.9%)	17 (29.8%)	11 (40.7%)
Ischemic Heart Disease (IHD)	100 (8.4%)	76 (18.8%)	12 (9.2%)
Migraine	102 (8.6%)	---	28 (21.4%)
Parkinson Disease	96 (8.1%)	48 (12.4%)	---
Multiple-Sclerosis	100 (8.4%)	37 (9.2%)	---
Traumatic Brain Injury (TBI)	100 (8.4%)	50 (12.4%)	1 (0.8%)
Stroke	198 (16.6%)	67 (16.6%)	44 (33.6%)


* p < 0.05 (between the distribution of patients included and not included on that sub-sample)

**Table 3:** Quartimin rotated loadings* of the Exploratory Factor Analysis with 7 Factors.

	1	2	3	4	5	6	7
**Understanding & Communicating. D1**							
concentrating	**0.54**	-0.26		-0.17	0.17	0.10	
remembering	**0.64**			-0.10	0.18	0.11	
finding solutions	**0.56**	-0.12	0.20		0.12	0.24	
learning new task	**0.64**				0.12	0.15	
understanding	**0.73**			0.10		0.15	-0.12
conversation	**0.50**					0.41	
**Getting around. D2**							
standing				**0.73**	0.28	0.12	
standing up	0.25	-0.35	0.10	**0.57**		-0.26	
moving around	0.15	-0.39	0.27	**0.41**	0.10		0.13
getting out of home		**-0.4**	0.35	0.20	0.25	0.23	0.19
walking	-0.16	-0.14		**0.59**	0.34	0.20	
**Self Care. D3**							
washing		**-0.66**		0.15			-0.34
dressing		**-0.79**		0.13			-0.19
eating		**-0.72**		-0.27	0.16	0.10	
staying by yourself	-0.20	**-0.44**	0.14			0.35	-0.17
**Getting along with people. D4**							
dealing with people unknown	0.24					**0.76**	
maintaining friendship	0.17					**0.69**	-0.17
getting along with people close	0.19		0.16	-0.17		**0.43**	
make new friends	0.19			0.10		**0.75**	
sexual activities		-0.20	-0.13	0.09	0.12	0.29	**-0.36**
**Life activities: household. D5.1**							
household responsibilities					**0.77**		-0.17
doing household tasks well					**0.90**		
doing housework needed					**0.86**		-0.11
household work done quickly	0.10			0.18	**0.84**		
**Life activities: work or school. D5.2**							
day to day work/school					0.11	0.11	**-0.81**
doing most important work well		-0.11					**-0.84**
getting work done needed							**-0.90**
getting work done quickly					0.14		**-0.78**
**Participation in society. D6**							
problems in communities	-0.10	-0.11	0.13	0.12	0.17	**0.47**	-0.29
problems because of barriers			0.14	**0.32**		0.27	-0.20
living with dignity			0.15	0.10		**0.26**	-0.17
time spend on health condition	-0.16	-0.13	**0.47**		0.39		
been emotionally affected			**0.55**	-0.11	0.15	0.21	-0.17
drain on financial resources	0.24	0.11	**0.52**	0.18	0.23		-0.29
problems for the family			**0.49**			-0.11	-0.36
problems doing things for relaxation			**0.32**		0.24	0.29	-0.11

*only factor loadings above 0.1 are shown.

The distribution characteristics and reliability coefficients of WHODAS-2 and SF-36 scores are reported in Table 4. The global WHODAS-2 mean score was 24.8(SD = 19.3), ranging from 0.0 to 93.5. The proportion of missing values was lower than 16% for most of the WHODAS-2 domains (with the exception of 'life activities: work or school', which was not responded by 50.2% of the sample). The floor effect was not relevant, but quite a high ceiling effect was present in almost all domains, especially for 'Self-care' (53.6%). Cronbach's alpha was above 0.7 for all WHODAS-2 scales, being the highest for the two domains of 'Life activities' and for the Global score (0.94-0.98). Last column of Table 4 shows the results on test-retest evaluation of reproducibility. The ICC was lower than Cronbach's alpha coefficient, but achieved the recommended standard of 0.7 for 4 of the domains.

**Table 4:** Distribution of scores and reliability coefficients for the WHODAS-2 and SF-36 domains

	Mean	SD	Observed Range	Missing domain (%)	Floor (%)	Ceiling (%)	Cronbach's alpha		ICC (n = 404)
**WHODAS-2**									
Understanding and Communicating	17.9	20.9	(0.0 - 100.0)	2.7	0.6	29.1	0.88		0.612
Getting Around	27.8	27.1	(0.0 - 100.0)	2.8	1.0	25.4	0.80		0.197
Self Care	14.4	21.2	(0.0 - 100.0)	2.8	0.3	53.6	0.77		0.524
Getting along with people	18.9	23.3	(0.0 - 100.0)	5.0	1.2	36.1	0.81		0.642
Life Activities: household	37.7	34.4	(0.0 - 100.0)	13.3	11.1	24.8	0.94		0.680
Life Activities: work or school	37.9	38.1	(0.0 - 100.0)	50.2	19.8	27.3	0.98		0.690
Participation in Society	28.1	21.0	(0.0 - 91.7)	5.2	0.0	10.9	0.82		0.693
Global	24.8	19.3	(0.0 - 93.5)	15.2	0.0	3.6	0.95		0.738
**SF-36**									
Physical Functioning	65.4	29.8	(0.0 - 100.0)	4.5	4.6	10.8	0.94^†^	0.88*	0.791
Role Physical	49.0	42.0	(0.0 - 100.0)	4.4	33.4	30.5	0.85^†^	0.96*	0.696
Role Emotional	60.3	41.0	(0.0 - 100.0)	5.0	22.6	43.2	0.87^†^	0.83*	0.556
Social Functioning	64.9	29.0	(0.0 - 100.0)	3.3	4.0	23.4	0.68^†^	0.78*	0.533
Mental Health	61.2	21.2	(0.0 - 100.0)	5.1	0.7	1.4	0.78^†^	0.87*	0.714
Bodily Pain	57.6	29.5	(0.0 - 100.0)	3.3	2.1	21.3	0.83^†^	0.88*	0.610
Vitality	50.9	21.8	(0.0 - 100.0)	5.0	1.8	0.8	0.84^†^	0.94*	0.684
General Health	53.4	21.3	(0.0 - 100.0)	5.6	0.9	0.5	0.82^†^	0.87*	0.759
PCS	42.0	11.2	(13.3 - 71.6)	8.0	---	---	---	---	0.782
MCS	43.5	13.1	(-0.2 - 72.8)	8.0	---	---	---	---	0.676

†SF-36 version 1 (TBI, IHD, Migraine, Parkinson, MS, stroke, musculoskeletal): n = 993

*SF-36 version 2 (bipolar y depression): n = 198

Table 5 presents the MTMM Matrix, where the correlations hypothesized as moderate (in bold) were confirmed. The global WHODAS-2 score was moderately correlated with most of the scores of the SF-36, with the main exception of 'Bodily pain', which presented quite low correlations with all the WHODAS-2 domains. The 'Participation in society' domain presented moderate to high correlations (0.4-0.6) with all the SF-36 dimensions. Moreover, moderate correlations not previously hypothesized were found between 'Life activities at work or school' and 'Social functioning' from the SF-36(0.5), and between 'Life activities: household' and three of the SF-36 dimensions, 'Physical functioning' (0.6), 'Social functioning' (0.48), and 'Role physical' (0.47).

**Table 5:** Multitrait-multimethod matrix. Pearson correlation coefficients between the WHODAS-2 and the SF-36 scores.

	WHODAS-2								
		Understanding & Communicating	Getting around	Self Care	Getting along with people	Life activities: household	Life activities: work or school	Participation in society	Global
**SF-36**	**Physical Functioning**	-0.13	**-0.75**	**-0.40**	-0.15	-0.61	-0.41	-0.55	-0.55
	**Role Physical**	-0.05	-0.46	-0.19	-0.03	-0.47	-0.34	-0.44	-0.36
	**Role Emotional**	-0.33	-0.42	-0.33	-0.31	-0.45	-0.45	-0.50	-0.53
	**Social Functioning**	-0.38	-0.49	-0.44	**-0.44**	-0.48	-0.50	**-0.63**	-0.65
	**Mental Health**	**-0.50**	-0.36	-0.42	-0.49	-0.38	-0.44	-0.54	-0.60
	**Bodily Pain**	-0.14	-0.38	-0.17	-0.05	-0.32	-0.174	-0.33	-0.29
	**Vitality**	-0.45	-0.45	-0.40	-0.42	-0.43	-0.36	**-0.51**	-0.57
	**General Health**	-0.38	-0.42	-0.33	-0.37	-0.35	-0.33	-0.49	-0.51

Correlations expected to be high or moderate are shown in bold type letter.

Pearson coefficients are negative because the two instruments scores, have the opposite direction.

**Figure 1:**
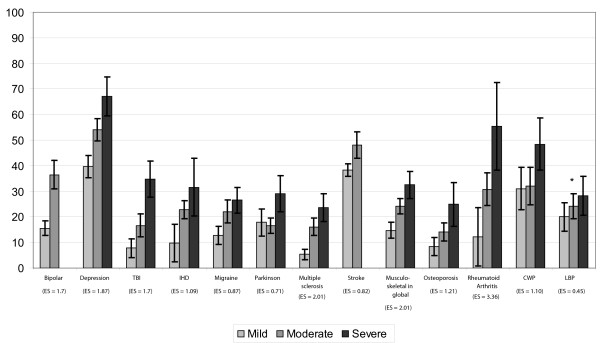
**WHODAS-2 global score for each severity group by pathology**. *no statistical significant difference. Mean and 95% confidence interval is shown. Effect Size (ES) coefficient among extreme groups.

The WHODAS-2 global score showed statistically significant differences among severity groups for all pathologies (Figure 1) with ES coefficients over 0.7 between mild and severe groups, except for low back pain. Table 6 shows mean scores of the specific domains by each severity group. Three of the WHODAS-2 domains (Getting along with people, Life activities household, and life activities work or school) presented non-significant differences among severity groups for more than half of the pathologies. For physical disorders, in general no significant differences across severity were observed in the understanding and communicating domain, and the ES coefficients were generally smaller than for the mental or neurological conditions. The results showed that at least 4 of the 7 WHODAS-2 domains differ statistically by severity groups for all conditions, except stroke. Most of the mean differences between extreme groups presented a ES coefficient > 0.5.

**Table 6:** WHODAS-2 domain specific scores by disorder, according to severity level.

Pathology (n)	Understanding & Communicating	Getting Around	Self Care		Getting along with people	Life Activities: household	Life Activities: work or school	Participation in Society
Bipolar	ES = 1.03	ES = 0.69	ES = 1.43		ES = 0.85	ES = 0.56	ES = 1.97	ES = 1.27
Eutimic (78)	21.4 (19.6)	9.0 (12.3)	4.1 (8.5)		19.8 (20.5)	20.1 (24.8)	15.8 (22.2)	15.3 (15.4)
No eutimic (36)	44.0 (26.5)*	20.7 (24.4)*	23.3 (20.4)*		38.9 (26.1)*	34.7 (28.8)*	72.9 (40.0)*	37.2 (20.6)*
Depression	ES = 1.44	ES = 1.24	ES = 1.82		ES = 1.61	ES = 0.56	ES = 0.75	ES = 1.5
Mild (36)	34.3 (20.1)	27.6 (21.9)	20.9 (21.5)		44.6 (23.1)	54.1 (30.4)	71.2 (38.2)	37.4 (15.0)
Moderate (30)	49.2 (19.5)	42.1 (24.5)	48.3 (22.0)		57.5 (28.9)	63.3 (33.3)	87.9 (18.5)	47.9 (13.3)
Severe (19)	66.8 (26.4)*	57.6 (27.7)*	60.5 (22.2)*		80.7 (21.2)*	72.1 (35.4)	94.9 (13.5)	61.6 (18.1)*
TBI	ES = 1.21	ES = 1.12	ES = 1.22		ES = 1.1	ES = 0.96	ES = 1.59	ES = 1.49
Mild (36)	6.9 (10.6)	4.2 (12.2)	1.7 (5.1)		8.3 (15.6)	10.8 (18.4)	10.3 (18.0)	9.8 (15.2)
Moderate(33)	17.6 (19.1)	9.7 (15.1)	4.8 (10.3)		15.9 (16.4)	18.6 (20.1)	25.0 (30.1)	22.0 (16.1)
Severe (31)	27.6 (22.2)*	31.1 (32.2)*	22.7 (24.4)*		31.1 (25.3)*	36.6 (34.0)*	61.2 (42.2)*	39.4 (24.0)*
IHD	ES = 0.89	ES = 1.21	ES = 1.09		ES = 0.44	ES = 0.89	ES = 0.69	ES = 1.28
Mild (12)	0.8 (2.9)	10.4 (19.8)	2.5 (8.7)		4.9 (6.6)	13.8 (35.0)	47.1 (50.4)	16.0 (13.9)
Moderate (71)	6.6 (9.5)	32.0 (29.0)	16.3 (18.6)		13.4 (12.3)	42.9 (43.0)	57.1 (43.1)	29.9 (16.9)
Severe (17)	11.5 (15.4)*	44.1 (32.4)*	26.5 (27.6)*		24.5 (24.2)*	63.3 (45.3)	52.4 (46.4)	37.3 (21.5)*
Migraine	ES = 0.57	ES = 0.54	ES = 0.36		ES = 0.51	ES = 0.97	ES = 0.61	ES = 1.07
Mild (27)	10.8 (9.3)	9.1 (13.1)	4.1 (9.3)		10.1 (10.4)	18.5 (20.7)	20.5 (17.4)	15.7 (10.7)
Moderate (29)	21.9 (16.7)	15.5 (20.1)	10.7 (17.5)		14.8 (19.8)	34.6 (23.6)	30.8 (19.4)	24.6 (13.5)
Severe (46)	22.4 (24.3)] *	23.2 (25.6) *	8.9 (16.6)		20.0 (24.6)	43.3 (29.5)*	34.8 (22.5) *	31.2 (16.3)*
Parkinson	ES = 0.67	ES = 1	ES = 1.05		ES = 0.54	ES = 0.07	ES = 0.48	ES = 0.91
Mild (13)	8.5 (10.1)	13.0 (20.6)	5.0 (8.7)		9.5 (11.4)	30.0 (22.4)	33.0 (32.8)	21.8 (13.8)
Moderate (54)	9.4 (9.2)	20.5 (20.6)	16.3 (19.5)		12.0 (12.7)	18.8 (22.1)	21.4 (19.9)	22.4 (15.2)
Severe (26)	19.8 (19.4)*	42.5 (33.0)*	31.9 (30.6)*		20.7 (23.8)	28.3 (27.5)	18.6 (28.4)	39.4 (21.4)*
Multiple sclerosis	ES = 0.42	ES = 3.08	ES = 1.34		ES = 0.48	ES = 1.22	ES = 1.11	ES = 1.71
Mild (43)	2.4 (5.9)	4.4 (8.9)	1.6 (5.7)		4.6 (9.5)	8.6 (13.4)	4.9 (9.8)	8.7 (9.8)
Moderate (36)	6.5 (9.1)	26.4 (17.7)	5.6 (12.1)		10.2 (11.7)	24.3 (19.9)	9.9 (16.8)	21.7 (14.9)
Severe (21)	5.2 (8.1)	55.4 (26.1)*	22.4 (26.1)*		9.8 (13.1)	27.4 (18.8)*	18.4 (15.9)*	33.5 (21.2)*
Stroke	ES = 0.22	ES = 0.85	ES = 0.6		ES = 0.49	ES = 0.53	ES = 0.22	ES = 0.47
Mild (78)	9.3 (12.2)	51.6 (24.4)	18.0 (20.8)		11.7 (16.7)	90.0 (21.7)	97.7 (12.1)	43.1 (19.6)
Moderate (24)	12.2 (15.9)	71.2 (18.0)*	32.4 (31.9)*		21.3 (27.3)	100.0 (0.0)*	100.0 (0.0)	50.8 (16.5)
Osteoporosis	ES = 0.49	ES = 1.44	ES = 0.53		ES = 0.7	ES = 1.1	ES = 0.3	ES = 1.31
Mild (41)	8.6 (11.8)	9.8 (16.1)	2.3 (11.4)		6.4 (11.2)	14.8 (24.2)	6.4 (13.7)	8.4 (13.3)
Moderate (27)	12.4 (13.3)	15.0 (11.6)	3.3 (6.2)		10.3 (14.6)	28.8 (19.0)	20.0 (19.8)	14.2 (10.9)
Severe (17)	15.6 (18.9)	35.7 (21.6)*	9.4 (17.5)		18.9 (28.3)*	42.7 (28.1)*	10.7 (15.2)	28.6 (19.8)*
Rheumatoid Arthritis	ES = 0.88	ES = 3.73	ES = 3.98		ES = 0.17	ES = 4.18		ES = 5.85
Mild (5)	15.0 (12.7)	11.3 (21.8)	6.0 (13.4)		15.0 (13.7)	20.0 (18.7)	0.0 (.)	10.0 (9.6)
Moderate (16)	27.8 (20.2)	33.2 (17.6)	16.3 (15.9)		18.8 (15.1)	47.3 (20.3)	35.7 (14.3)	28.6 (13.8)
Severe (2)	30.0 (28.3)	84.4 (4.4)*	60.0 (14.1)*		12.5 (17.7)	90.0 (0.0)*	---	62.5 (5.9)*
Chronic Widespread Pain (CWP)	ES = 1.23	ES = 0.69		ES = 0.62	ES = 0.47	ES = 0.13	ES = 0.90	ES = 1.18
Mild (8)	22.7 (12.8)	36.5 (18.0)	8.7 (14.1)		27.8 (21.4)	54.2 (21.9)	40.2 (20.5)	32.6 (17.0)
Moderate (21)	27.1 (20.4)	45.0 (22.9)	13.6 (16.5)		25.7 (24.3)	55.7 (27.1)	30.6 (20.9)	28.9 (15.5)
Severe (13)	47.3 (26.1)*	48.6 (17.0)	19.2 (20.2)		38.7 (24.7)	57.7 (31.7)	58.2 (19.5)	53.8 (18.7)*
Low Back Pain (LBP)	ES = 0.03	ES = 0.56	ES = 0.62		ES = 0.13	ES = 0.47	ES = 0.37	ES = 0.87
Mild (42)	15.6 (18.3)	27.2 (23.6)	7.4 (12.7)		12.7 (19.3)	31.5 (29.6)	22.2 (34.2)	20.5 (18.8)
Moderate (44)	22.2 (19.8)	32.5 (20.8)	13.4 (16.1)		14.4 (17.1)	35.1 (26.2)	26.8 (32.0)	28.3 (21.0)
Severe (24)	15.1 (16.5)	40.4 (23.6)	17.5 (21.1)*		10.5 (14.5)	45.2 (27.9)	34.9 (34.5)	39.3 (25.9)*


Effect size (ES) coefficient among the extremes groups

* p < 0.05

**Figure 2:**
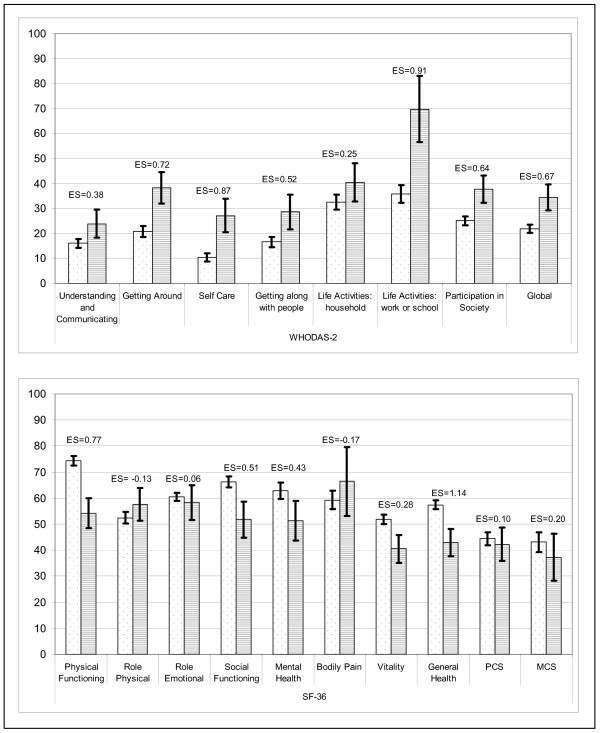
**WHODAS-2 scores for patients working (dots) and not working-sick leave (striped)**. Mean and 95% confidence interval is shown. Effect Size (ES) coefficient between working and not working-sick leave patients.

Almost all the WHODAS-2 scores showed statistically significant differences (p < 0.001) between working patients and those not working due to ill health (Figure 2), and all except 2 presented an ES above 0.5. For the SF-36 scores, only 3 out of 10 ES coefficients were moderate or high.

**Figure 3:**
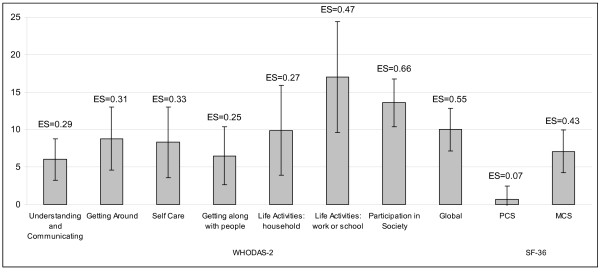
**Mean chage of the WHODAS-2 scores and the SF-36 component summaries, after 3 months**. Mean change and 95% confidence interval is shown. Effect Size (ES) responsiveness coefficient.

Figure 3 shows the mean change of the WHODAS-2 scores and SF-36 component summaries among the subsample of patients that had improved. The ES coefficients were moderate for 2 WHODAS-2 domains: 'Life Activities: work or school' (ES = 0.47), and 'Participation in Society' (ES = 0.66); and for the Global score (ES = 0.55). For the rest of the scores the ES was less than 0.4.

## Discussion

This study confirms the conceptual model of the WHODAS-2, which has shown good metric properties among patients with chronic conditions in Europe in the MHADIE project: a very high reliability, good ability to discriminate among known groups and adequate capacity to detect change over time. Therefore, these results support the adequacy of the WHODAS-2 to measure disability in a wide range of physical and mental disorders.

The goodness of fit indices obtained with the CFA models together with the high factor loadings confirmed the 7 domain structure of WHODAS-2 and the global score [44], as proposed by developers. Only some concerns should be raised. The RMSEA wasn't below the standard as recommended. CFA modification indexes (data not shown) suggested that the structural model behind data may be improved if some items from 'Participation in Society' domain were relocated on some of the other factors. Nonetheless, accepting the original structure proposed by developers would improve comparability with past and ongoing WHODAS-2 studies. Therefore, we suggest using the structure of the WHODAS-2 as it is now known, taking into account the expert-based validity criteria originally applied and that, despite the described concerns, our findings confirmed it on a heterogeneous sample. Moreover, the structure is quite consistent with previous results, both from specific populations[23,24] and from the modified version[25].

The low proportion of missing values suggests the easy completion for a wide range of patients, indicating the high feasibility of WHODAS-2. A great percentage of missing data was only found at the domain of activities at work or school (50.3%), which is clearly related with the proportion of respondents neither working nor being students. The moderate percentage of patients with the best possible score in several domains suggests the possible unsuitability of the WHODAS-2 to differentiate among very low grades of disability. This may not be a limitation for measuring disability on patient samples, but one should be cautious when using it on other samples such as general population, which has earlier shown a very high ceiling effect[26]. Nonetheless, the distribution of the 'Participation in society' score merits a comment. No patient has the worst possible score (floor effect) and presents the lowest ceiling effect (11%), indicating that this domain is able to characterize a wide range of scenarios and is perhaps reflective of the final common pathway in which disability is manifested in the societal context.

The high internal consistency coefficients indicate good reliability. All of them were above the standard proposed for group comparisons (0.7) [51], which is consistent with findings from previous studies[23,15,19,21,22,24]. It is also remarkable that internal consistency coefficient for the global score reaches the most strict standard recommended for individual comparisons of 0.95. Reproducibility was acceptable, with the exception of the 'Getting around' domain (ICC = 0.19). Due to the long test-retest period, patient's mobility may have improved or worsened over 6 weeks, even though disease severity did not change substantially. The only study in which stability of the WODAS-2 has been assessed, presented excellent ICC coefficients (0.82-0.96) on patients with inflammatory arthritis[15].

The WHODAS-2, as designed for covering disability, measures the restrictions on daily life activities and social participation, while the Short form-36 Health Survey addresses patients' physical and mental health. The moderate magnitude of the associations among the two instruments is reflecting how the WHODAS-2 and the SF-36 measure different aspects of related concepts (disability and HRQL, respectively). In fact, coefficients found in previously published studies[23,15-18,20,21] were fairly similar to ours. These findings support the validity of WHODAS-2 to measure disability and its use as an outcome which complements HRQL.

The WHODAS-2 is able to detect differences between clinical-severity groups. Those patients classified as severe reported worse disability scores than mild patients, with a large difference for most of the health conditions (66%), and a moderate difference for 25% of them. Poor discrimination ability among severity groups were found only for 3 of the WHODAS-2 domains ('Getting along with people', 'Life activities household' and 'Life activities work or school'). Beside this, the instrument detects differences between patients who were working at the time of the study and those who were not working due to their health condition. This is the first time that such an ability is evaluated on the WHODAS-2, and is specially remarkable when talking about disability, probably more than being able to differentiate among severity groups (which has also been shown in other studies[15,16,22,23]).

Coefficients of change at 3 months were moderate or low for all domains. However the WHODAS-2 sensitivity to change may be under-estimated in our study due to the MHADIE patients' characteristics and design, such as the chronic profile of the conditions, and not being an evaluative intervention study. Moreover, this pattern of low improvement, also presented by the SF-36 (no physical change and moderate mental improvement), an instrument which has extensively demonstrated good responsiveness[52,21], is indicating the lack of a real great improvement in our sample rather than a problem of WHODAS-2 to detect change over time. In fact, a previous study has demonstrated how the WHODAS-2 is quite responsive (ES = 0.65) when change is measured after starting a treatment[21].

This study's results should be interpreted taking into account some limitations. Firstly, the study was not specifically designed for evaluating responsiveness, since the optimum design for this should include an intervention which would produce a clear improvement or an event closely related to deterioration. However, assuming that a change in severity would be accompanied by a change in self-perceived disability, patient improvement was measured indirectly due to the lack of a gold standard for disability change. Secondly, the interval for test-retest evaluation is longer than the standard period used to assess reproducibility. However, the selection strategy applied assured the needed stability and ICC coefficients showed agreement between evaluations. Moreover, it should be noted that different WHODAS-2 linguistic versions have been administered regarding the country setting, but analyzed as a whole. To test the equivalence of these versions, differential item functioning (DIF) analysis would be required [53]. However, it was not possible in our study because of the sample design, where most of the health conditions were recruited only in one country, making impossible to differentiate the effect of these two variables. Finally, other minor limitations are related to version differences. The SF-36 v2 was used for Spanish patients with psychiatric disorders but, as version 1 and 2 of the SF36 are quite similar, no impact on results was expected. On the other hand, proxy versions used on those patients unable to respond were negligible.

## Conclusions

Despite some limitations, as discussed above, the results provide considerable support to the WHODAS-2 utilization as a common, international, and interdisciplinary instrument to measure disability. Furthermore, it is of special relevance because of being the only measure based on the ICF biopsychosocial model. A strength of the study is that the underlying latent structure originally designed by developers has been confirmed for the first time. This has moreover been conducted on an heterogeneous sample (different health conditions in several European countries), which gives even higher worth to results, together with the assessment of its good metric properties. In conclusion, the WHODAS-2 is adequate to evaluate disability in patients with chronic conditions, which may help to eliminate barriers on developing policies, giving excellent evidence of these populations' needs.

## Abbreviations

(CFI): Comparative Fit Index; (CWP): Chronic widespread pain; (EFA & CFA): Exploratory and Confirmatory factor analyses; (ES): Effect Size coefficient; (HRQL): Health Related Quality of Life; (ICC): Intra-class Correlation Coefficient; (ICF): International Classification of Functioning, Disability, and Health; (IHD): Ischemic heart disease; (LBP): Low back pain; (MHADIE): Measuring Health and Disability in Europe: Supporting policy development; (MTMM): Multitrait Multimethod; (PCS & MCS): Physical and Mental Components Summaries; (RMSEA): Root Mean Squared Error of Approximation; (SD): Standard Deviation; (SF-36): The Short Form-36 Health Survey; (TLI): Tucker-Lewis Index; (TBI): Traumatic brain injury, (WHO): World Health Organization, (WHODAS-2): World Health Organization Disability Assessment Schedule 2.0.

## Competing interests

There are no conflicts of interest with respect to the submitted data, except for EV, who would like to state that he has received research grants, and served as consultant, advisor or speaker for the following companies: AstraZeneca, Bristol-Myers Squibb, Eli Lilly, Forest Research Institute, GlaxoSmithKline, Janssen-Cilag, Jazz, Lundbeck, Novartis, Organon, Otsuka, Pfizer Inc, Sanofi-Aventis, Servier, and UBC.

## Authors' contributions

All listed authors participated meaningfully in the study and that they have seen and approved the final manuscript. Authors' contributions were:

OG conceptualized and oversaw analyses, and wrote the article.

JA and GV contributed and revised statistical analyses and contributed to the interpretation of data.

SC contributed to the conception, design and writing of the article.

J L AM, MN, AC, OS, HB, VR, CF, EV, NK, AR, ML, designed the study, recruited the patients, oversaw all aspects of the study implementation, and contributed to the writing of the article.

JA contributed to the interpretation of data and oversaw all aspects and reviewed the article for important intellectual content.

MF oversaw all aspects, contributed to the conception and design of the article, contributed to the statistical analyses, carried out the interpretation of data, and contributed to the writing of the article.
